# mtDNA and Mitochondrial Stress Signaling in Human Diseases: A Special Issue

**DOI:** 10.3390/ijms21072617

**Published:** 2020-04-09

**Authors:** Vito Pesce, Angela Maria Serena Lezza

**Affiliations:** Department of Biosciences, Biotechnologies and Biopharmaceutics, University of Bari, Via Orabona, 4, 70125 Bari, Italy; vito.pesce@uniba.it

The completion of the Special Issue dedicated to “mtDNA and mitochondrial stress signaling in human diseases” requests a final overall look to highlight the most valuable findings among the many presented data. 

Mitochondrial DNA (mtDNA) has gained a growing relevance since its discovery as the multiple roles performed by this cytoplasmic genome, unique to animal cells, have been unveiled. The sequencing of human mtDNA has demonstrated the need for a coordinated expression between the nuclear and the mitochondrial genomes in order to assemble functional mitochondrial respiratory complexes that allow oxidative phosphorylation to be carried out to obtain ATP. The close dependence of some energy-consuming tissues, such as those of the central nervous system and skeletal muscles, on the ATP produced inside the organelles has led to the identification of several mtDNA mutations responsible for the onset of devastating diseases known as mtDNA-related mitochondrial pathologies. One of the prime and unanimously acknowledged scientists working on these diseases is the leader of the large “mitochondriacs” group at Columbia University, Dr. S. DiMauro, who contributed to the Special Issue his very personal “A Brief History of Mitochondrial Pathologies” [1]. In his paper, both mtDNA-related and nuclear DNA-related pathologies were described, focusing attention on the complex interplay between the nucleus and mitochondria. A deepened knowledge of such diseases pinpointed the importance of the mtDNA sequence specifically present in cells and tissues and of the polyploidy of the molecule that is the coexistence, in the same cell, of multiple copies of mtDNA that might (homoplasmy) or might not (heteroplasmy) share an identical sequence. The complicated issue of the “threshold effect”—the dependency of the phenotypic manifestation of the disease on the ratio between mutated and wild-type mtDNA molecules inside each cell [1]—has driven attention to the possibility that different mtDNA sequences might coexist in the same cell without overt pathological consequences. In fact, the high mutation rate of mtDNA [2] facilitates the occurrence of sequence mutations that might have no evident effect on the structure and/or metabolism of the organelles, but might confer some kind of sensitivity or predisposition that we are currently not able to fully evaluate. This is the reason for studies focusing on mtDNA sequence polymorphisms, which might have neutral or dangerous effects for the organelles and/or the whole cell. An example of such analysis is presented in the paper by Hirose et al. [3], dealing with a natural polymorphism in the *cytochrome b* gene that induced, in mouse carriers, alterations in mitochondrial activities and body composition and metabolism. In particular, carriers presented middle-age obesity, a susceptibility to diet-induced obesity—as well as to age-related inflammatory disease—and an alteration of their gut microbiota that might also make the host more sensitive to metabolic and inflammatory disorders. Along the same line of mtDNA polymorphisms conferring some kind of susceptibility to specific diseases is the constantly increasing list of mtDNA variants characterized in human cancers [4]. Such mutations can be present at homoplasmy or heteroplasmy, and their effects have not been completely explained. On the other hand, human cancer-derived cell lines are useful for preclinical studies and for tests on drug toxicity and drug discovery. In particular, the heteroplamic new mtDNA polymorphisms, described in the paper about the commonly used HepaRG and SJCRH30 cell lines [5], might be useful for following mtDNA maintenance along cell line passages in various experimental designs.”. Furthermore, future sequencing studies on mtDNA from tumors and control tissues might help to elucidate if the reported variants are rhabdomyosarcoma (SJCRH30)- and hepatocarcinoma (HepaRG)-specific changes.

The existing tight and intricate interplay between the nucleus and mitochondria has been deeply characterized through three original studies analyzing the different components of such relationships, such as autophagy, mtDNA content and damage, mitochondrial structure and metabolism. In the paper by Green et al. [6], the cell responses evoked by a mutant form of the nuclear gene for the TERT catalytic constituent of telomerase—competent in telomere elongation, but not in mitochondrial localization—were examined. The absence of TERT from the mitochondria induced increased autophagy in cells challenged with oxidative stress, whereas the restoration of the regular subcellular localization blunted the autophagic response under oxidative stress. The study pointed out that the missing mitochondrial localization of TERT might be a signal impinging on various responses, including that to oxidative stress, to adapt the whole cell to this absence. A similar kind of retrograde signaling, deriving from the absence of specific mtDNA repair enzymes can be envisioned in the study by Chimienti et al. [7]. The ablation of the nuclear gene for the mitochondrial isoform of the 8-oxoG DNA glycosylase/apurinic or apyrimidinic (AP) lyase (OGG1) or the endonuclease III homolog (NTH1) affected mtDNA. In fact, a common feature of both knockout mouse strains was the increase in mtDNA content, suggestive of a compensatory response to the oxidative damage of mtDNA molecules. Other experimental approaches utilized in the study have allowed the verification of the idea that the mtDNA region encompassing the origin of replication of the H-strand and the transcription promoters of both strands, namely the D-loop region, is a hotspot for oxidative damage. Thanks to these findings, it was possible to suggest that mtDNA alterations could also be somehow communicated to the nucleus (retrograde communication) and impinge on the regulation of nuclear expression, leading to the proliferation of mtDNA molecules. Another good example of the tight interrelationships between the mitochondria and nucleus is provided in the paper by Suzuki-Hatano et al. [8] that scrutinized some features of the mitochondrial disease Barth syndrome, caused by mutations in the nuclear gene for tafazzin (TAZ), which is responsible for the remodeling of the mitochondrial phospholipid cardiolipin. Structural (increased mitochondrial fragmentation and decreased mtDNA content) as well as metabolic (reduced ATP generation and oxygen consumption, and increased ROS production) features of patients fibroblasts’ organelles were affected by the mutations and were retrieved by adeno-associated virus (AAV) transduction with the TAZ wild-type gene. The novelty of a possible gene therapy through AAV-mediated delivery to patients’ tissues thus enlivened the reported alteration of a mitochondrion-specific lipid, able to evoke changes in nuclear expression and to affect mitochondrial biogenesis and activity. The Special Issue also includes a large number of studies dealing with signaling of mitochondrial stress, mainly oxidative stress, not due to single gene mutations but associated with multigenic diseases such as diabetes, Parkinson’s disease (PD) or celiac disease (CD), or to physiological aging. A paper showed the higher prevalence of heteroplasmic mtDNA mutations, mostly localized in the so-called control region, in diabetes patients affected by diabetic retinopathy (DR). The sequential suggestion that mtDNA damage might contribute to the onset of DR and might represent a potential therapeutic target through strategies for the removal of mutated circulating molecules completed this interesting work by Malik et al. [9]. As for neurodegenerative diseases associated with mitochondrial oxidative stress, a paper dealt with a *C. elegans* model of Parkinson’s disease (PD), highlighting the relevance of environmental interactions with genetic deficiencies [10], and another focused on the possible use of mitochondrion-derived vesicles as early biomarkers of PD [11]. The study by Hartman et al. [10] unveiled the interactions between the presence of mutations in genes involved in mitochondrial dynamics (fission and fusion) and exposure to UVC or to the neurotoxicant 6-hydroxydopamine (6-OHDA), leading to the opposite effects of an increased sensitivity to UVC-induced neurodegeneration or of an increased protection from 6-OHDA-induced neurodegeneration in worm dopaminergic neurons. On the other hand, mutations in mitophagy genes (PINK-1 and PRKN homologs) led to a generally increased sensitivity of neurons to damage, with the exception that *pink-1* mutants were markedly protected from 6-OHDA-induced neurodegeneration. The overall message was that genetic deficiencies in mitochondrial dynamics and mitophagy markedly impact on the sensitivity of *C. elegans* to environmentally-induced neurodegeneration and that interactions between the genes for these mitochondrial functions and the environment might contribute to PD onset. The paper by Picca et al. [11] focused on the possibility of using small extracellular vesicles (sEV)/exosomes loaded with mitochondrion-derived molecules and secreted into body fluids as early candidate biomarkers for PD development. In fact, the content analysis of such loaded cargos might allow the following up of the association between mitochondrial dysfunction—particularly as for the disruption of mitochondrial quality control—and systemic inflammation, featuring the disease progression. The description of the purification and characterization protocol for sEVs, which could be used only for associative-type studies, opened, however, an intriguing possibility as to their application for diagnostic and therapeutic purposes. In the group of diseases associated with mitochondrial oxidative stress is included CD, as a recent study has demonstrated a compensatory mitochondrial proliferation in CD patients’ lymphocytes in response to the oxidative stress and pro-inflammatory conditions characterizing the disease [12]. In order to deepen the knowledge of mitochondrial involvement in gluten-related disorders, an in vitro study about the effect of gliadin on mtDNA content and damage, as well as on mitochondrial biogenesis in Caco-2 cells, was performed [13]. The gliadin-induced oxidative stress evoked a compensatory response, demonstrated by increases in the expression of mitochondrial biogenesis proteins and in mtDNA content, showing a slightly slower and longer response with respect to mtDNA content than to the expression of the proteins. The paper also assessed the kinetics of the mtDNA damage that occurred very early on at all three analyzed regions—namely the D-loop, Ori-L and ND1/ND2 regions—and showed that the D-loop was a more fragile target, in terms of both its degree of the damage and its persistence, confirming, in this original system, the results obtained in other experimental models [14,15]. Mitochondrial oxidative stress is also acknowledged as a feature of the aging process, but much less is known about its presence in those individuals, humans or animals, naturally able to reach extended longevity. The study by Chimienti et al., [16] analyzed different mitochondrial biogenesis-related parameters with a particular focus on mtDNA damage in livers from aged and extremely aged rats, finding some novel and interesting results. In fact, the only relevant differences between the two age groups were the decreases in the amount of TFAM-bound mtDNA and the incidence of oxidized purines at all assayed regions, in the extremely aged rats with respect to their aged counterparts. A correlation between the incidence of oxidized purines and TFAM-bound mtDNA amount was found only in the regions encompassing the mtDNA replication origins of the aged rats, but not in the counterparts of the extremely aged rats. This led to the suggestion that a different, fine-tuned regulation of TFAM binding existed in the two age groups, implying different paces of aging and extended aging as concluded in a previous work dealing with other mitochondrial markers [17]. Among the most common age-related diseases is age-related macular degeneration (AMD), whose cause is not known, but in which oxidative stress-related damage to retinal pigment epithelium (RPE) is an early event. The review by Kaarmiranta et al. [18] focused on mtDNA oxidative damage, likely caused by the age-related mitochondrial oxidative stress, as a major trigger of AMD pathogenesis. For this purpose, the paper examined a wide list of mtDNA-damaging agents, distinguished as ROS-inducing agents and ROS-independent agents. The common cell response to increased mtDNA damage should be a counteracting reaction carried out through increased repair processes and the slowing down of damage progression. The latter is presently pursued by ROS-targeting AMD therapies, while an increase in the mtDNA repair processes is a distant goal, as knowledge of the mtDNA repair mechanisms and of their regulation still requires a large amount of study. In the Special Issue is included another review [19] dealing with the potential beneficial effect of thyroid hormone (TH) treatment in the mitochondrial oxidative stress induced by cardiac ischemia-reperfusion (IR) and heart failure. Because of the large morbidity of heart diseases in Western countries and the relevant role of oxidative stress-related mitochondrial dysfunction in these pathologies, research about agents able to restore mitochondrial efficiency is constantly proceeding. In particular, mitochondrial quality control (MQC) has gained wide attention, since it balances repair and elimination of damaged molecules and organelles. TH treatment, in pathological heart models, has recently shown the ability of these hormones to act as cardio-protective agents by regulating different mechanisms of MQC, leading to the increased turnover of damaged mitochondria and the restoration of organelle bioenergetics with their replacement through biogenesis. TH might thus couple MQC and biogenesis, and these were examined in the review as potential contributors to therapies for the mitochondrial component of heart diseases. The high number of different experimental models and diseases analyzed in the present Special Issue should permit a broad and updated view of this fast-moving scenario, highlighting the involvement of mtDNA in multiple cases as the origin or target of the oxidative stress situation and always as a participant in the bidirectional nucleus-mitochondria communication that controls many crucial aspects of cell metabolism and life.
